# Patterns of change in the population and spatial distribution of oriental white storks (*Ciconia boyciana*) wintering in Poyang Lake

**DOI:** 10.13918/j.issn.2095-8137.2016.6.338

**Published:** 2016-11-18

**Authors:** Zhen-Hua WEI, Yan-Kuo LI, Peng XU, Fa-Wen QIAN, Ji-Hong SHAN, Xiao-Bin TU

**Affiliations:** ^1^ College of Life Sciences, Jiangxi Normal University, Nanchang 330022, China; ^2^ Key Laboratory of Forestry Protection of State Forestry Administration, Research Institute of Forest Ecology, Environment and Protection, Chinese Academy of Forestry, Beijing 100091, China; ^3^ Wildlife Service of Jiangxi Province, Nanchang 330038, China

**Keywords:** Oriental white stork, Poyang Lake, Population size, Spatial distribution

## Abstract

Using total counts in simultaneous annual surveys, we monitored the population size and spatial distribution of oriental white storks (*Ciconia boyciana*) wintering in Poyang Lake between 1998 and 2011. Results showed that Poyang Lake wetland is an important wintering ground for oriental white storks, with an annual average population number of 2 305±326. The population sizes in 2004, 2005, 2010, and 2011 were higher than the highest-ever estimate of its global population. In 2005, we recorded 3 789 individuals, which was the maximum population number within the period of 1998–2011. The storks inhabited 52 lakes, with the greatest distance between these lakes being 180.3 km. The storks presented a clustered distribution pattern in the Poyang Lake wetland, irrespective of the number of individuals or occurrence frequencies. Shahu, Dahuchi, Banghu, and Hanchihu were most frequently used lakes and had the largest annual average numbers of storks. There was a significant positive correlation between occurrence frequency and annual average number of storks in the lakes. Most of the lakes important for storks were covered by existing nature reserves, though some lakes outside the reserves were also frequently used. About 64.9%±5.5% of the storks were found in nature reserves. In addition, the storks more frequently used and clumped in significantly larger flocks in lakes within nature reserves than lakes outside.

## INTRODUCTION

The oriental white stork (*Ciconia boyciana*) is a large migratory wader belonging to the genus *Ciconia*, family Ciconiidae, and order Ciconiiformes. It breeds primarily in the Amur and Ussuri basins along the border of Russia and China, and in northeastern China (BirdLife International, 2013; Liu & Li, 2008; Smirenski, 1991). Its main wintering grounds are in the lower Yangtze basin and southern China, including Taiwan and Hong Kong, with small numbers wintering in the Korean peninsula, Japan, the Philippines, northeastern India, Myanmar, and Bangladesh (BirdLife International, 2013). With a population size of only about 3 000 (Birdlife International, 2013), this species is listed as endangered on the IUCN Red List of Threatened Species, and is also included in Appendix Ⅰ of the Convention on International Trade in Endangered Species (CITES). In China, it is also listed as a level Ⅰ wild bird under special state protection (China Wildlife Propagation Institution for Protection, 1989).

The Poyang Lake wetland is an important wintering ground for the oriental white stork. Field surveys on its wintering population have been conducted by Chinese ornithologists since the 1980s, with the first report of 200 individuals observed in Poyang Lake presented in 1983 (Wu, 2002). This species and specific population has attracted increasing attention, especially after the establishment of the Poyang Lake Nature Reserve. Ji et al. (2006) observed 1 258 individuals by aerial survey across Poyang Lake in the winter of 2006, which accounted for 42.0% of its global population. In the winter of 2008, 3 909 individuals were observed in Poyang Lake, which was the highest-ever recorded global population (Wu et al., 2010). Wu et al. (2010) also noted more than 300 individuals-exceeding 10.0% of its global population-in each of 11 inner lakes of the Poyang Lake wetland.

Until now, few studies on oriental white storks covering the entire Poyang Lake wetland have been conducted due to the numerous inner lakes within the area. Currently, all available reports are confined to the Poyang Lake National Nature Reserve (PLNNR). In this study, we monitored the population size and distribution of oriental white storks across 77 inner lakes of the Poyang Lake wetland to better understand the population dynamics and identify important sites for the stork within the area.

## MATERIALS AND METHODS

### Study area

Poyang Lake (E115°49′-116°46′ and N28°11′-29°51′) is the largest freshwater lake in China, covering a drainage basin of 162 000 km^2^. It is in the north of Jiangxi Province and on the south bank of the Yangtze River. It has a subtropical humid climate with a distinct seasonal shift, characterized by an average annual precipitation of 1 636.4 mm and a mean annual temperature of 17-17.8℃. Poyang Lake receives runoff primarily from five tributaries, namely the Ganjiang, Xiuhe, Fuhe, Xinjiang, and Raohe rivers, and discharges into the Yangtze River in the north; however, reversed flow sometimes occurs due to an elevation of the Yangtze River water level. The Poyang Lake water level shows obvious seasonal fluctuation and can be divided into wet (April to September) and dry seasons (October to March) (Wang, 2004; Ye et al., 2011). In the wet season, the floodplains are inundated and form a large lake with a surface area of more than 3 000 km^2^. In the dry season, the lake shrinks to less than 1 000 km^2^ and forms a narrow meandering channel surrounded by numerous individual, dish-shaped inner lakes (Wang, 2004). 

The Poyang Lake wetland is an internationally important wintering ground for migratory waterbirds. About 310 bird species, including 155 winter visitors and 107 summer visitors, inhabit Poyang Lake. It has been estimated that over 98.0% of the global population of Siberian cranes (*Grus leucogeranus*), 80% of oriental white storks, 50% of white-napped cranes (*G. vipio*), and 50% of swan geese (*Anser cygnoides*) winter in Poyang Lake (Wu, 2002). Poyang Lake is also an important wintering ground for the hooded crane (*G. monacha*), common crane (*G. grus*), tundra swan (*Cygnus columbianus*), and Eurasian spoonbill (*Platalea leucorodia*). Nowadays, there are three nature reserves dedicated to the protection of migratory waterbirds, namely PLNNR, Jiangxi Poyang Lake Nanjishan Wetland National Nature Reserve (NWNNR), and Duchang Provincial Nature Reserve for Migratory Waterbird Conservation (DPNR).

### Bird census

**Figure 1:**
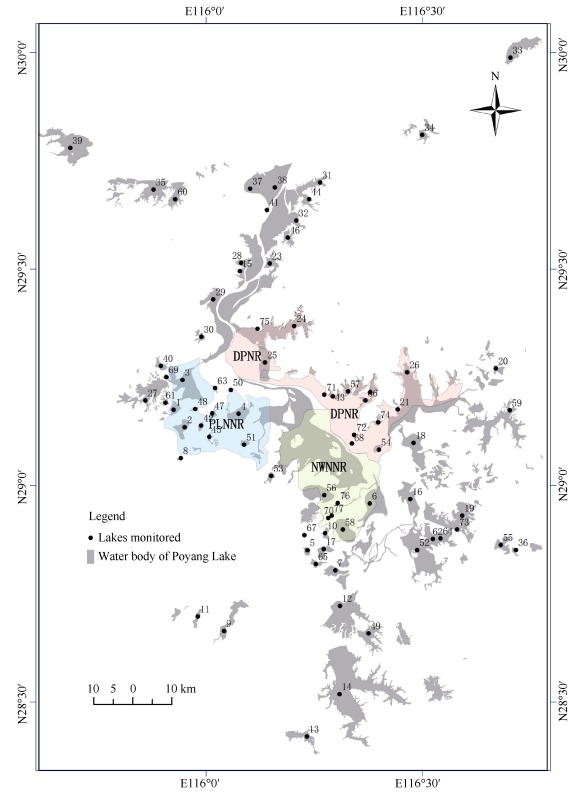
Map showing locations of the lakes involved in monitoring the wintering population of the oriental white stork in the Poyang Lake wetland

We monitored the population size and distribution of oriental white storks across 77 lakes of the Poyang Lake wetland (Figure 1). The study area covered most of the inner lakes in all 13 counties of Poyang Lake, including Nanchang, Xinjian, and Jinxian-which are part of the Nanchang municipality-Gongqingcheng, Ruichang, Duchang, Xingzi, Hukou, Pengze, Jiujiang, and Lushan District-which are part of the Jiujiang municipality-and Yugan and Poyang in the Shangrao municipality.

Each winter, from 1998 to 2011, we used total counts to investigate the number of oriental white storks across the 77 lakes on the same day to avoid repeats or omissions due to the frequent movement of the storks. Surveys were conducted in December or January in most years, except for 2009 and 2010 when the surveys were conducted in February. Forty survey groups were organized each year; each group was comprised of one or two professionals and one guide, equipped with monoculars (25-75×82) and binoculars (10×56). The survey members came from the Administration of PLNNR, NWNNR, and DPNR, Jiangxi Normal University, Jiangxi Agricultural University, Jiangxi Academy of Forestry, and local volunteers. Each year, investigators undertook a two-day training program on waterbird survey methods before the formal field survey. Each group began its land survey in the morning. If some sites were not reachable by foot, a boat was used. Whenever the investigators encountered oriental white storks, they stopped to observe, count, and record the number.

### Data analysis

To investigate whether significant changes in population numbers had occurred in Poyang Lake, we chose the population size of oriental white storks as the dependent variable and time (year) as the independent variable. The occurrence frequency and average number of storks in each lake were used to evaluate the importance of lakes to the storks. We also interpolated a raster surface from lake importance using an inverse distance weighted (IDW) technique to illustrate stork distribution (Tang & Yang, 2006). The generated raster size was 0.05°×0.05°. To compare the frequency of lake use under different protection statuses, we used a one-sample Kolmogorov-Smirnov test to examine whether the data were normally distributed. If the distribution was normal, analysis of variance (ANOVA) was implemented; if not, two independent sample tests were used to examine whether there was a significant difference in use intensity between protected and unprotected lakes. 

## RESULTS

### Population size

**Figure 2:**
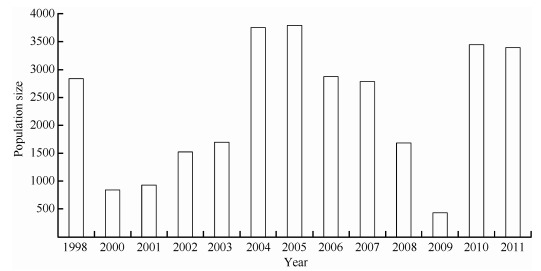
Dynamics of the oriental white stork population in Poyang Lake between 1998 and 2011

Between 1998 and 2011, the average population size of oriental white storks wintering in Poyang Lake was 2 305±326. Population numbers did show considerable fluctuations, but no significant patterns of change (Figure 2). There were 2 832 storks in 1998, but only 838 in 2000. The population then showed a significant exponential increase between 2000 and 2005 (R^2^=0.935, F=57.209, *P*=0.002), and reached its maximum number of 3 789. After 2005, the population number decreased linearly (R^2^=0.939, F=46.407, *P*=0.006) to the minimum number of 430 in 2009. The population increased sharply to 3 445 in 2010, and remained at a similar size in 2011. The numbers of oriental white storks in Poyang Lake in winter 2004, 2005, 2010, and 2011 were higher than the highest-ever estimate (3 000) of the global population.

### Spatial distribution

In the Poyang Lake wetland, 52 sub-lakes were used by wintering oriental white storks (Figure 1). The greatest distance between these lakes was 180.3 km. The numbers of oriental white storks in 32 sub-lakes exceeded 1% of the global population (Table 1). The maximum numbers of oriental white storks in each of the 32 lakes over the 14-year period, in descending order, were: Shahu, 1 770; Chengjiachi, 1 738; Dahuchi, 1 559; Nanhu, 1 510; Dachahu, 1 155; Banghu, 1 011; Zhonghuchi, 790; Sanhu, 686; Hanchihu, 674; Yufeng, 600; Candouhu, 566; Zhuhu, 565; Changhu, 531; Dalianzihu, 450; Xianghu, 328; Meixihu, 303; Linchonghu, 259; Liuhuachi, 210; Xieshanhu, 200; Sanniwan, 198; Qihu, 197; Dawuhu, 135; Zaohu, 121; Junshanhu, 114; Donghu, 92; Xihu (Nanchang) 91; Luojiaohu, 90; Beiganghu, 90; Zhushihu, 86; Nanshanhu, 85; Zhanbeihu, 80; Linghu, 62; Meixiihu, 56; Nanjianghu, 55; Changhuchi, 54; Chenjiahu, 37; Taipohu, 32; and Xihu (in Duchang county), 30. The average flock size of oriental white storks in these lakes was 286±34. The lakes where the number of oriental white storks exceeded 40.0% of the global population (i.e., 1 200) included Chengjiachi in Yugan county (1 738 individuals, winter 2004), Dahuchi in PLNNR (1 277, winter 1998; 1 559, winter 2011), and Shahu in PLNNR (1 770, winter 2007). 

**1:** Lakes with an oriental white stork population exceeding 1% of the global population

Lake	Year													Average
	1998	2000	2001	2002	2003	2004	2005	2006	2007	2008	2009	2010	2011	
Shahu							825	336	1 770				960	973
Chengjiachi						1 738								1 738
Dahuchi	1 277	294	261	43	325	440				368			1 559	571
Dachahu		69					303	108	93			1 155		346
Banghu			159				347	93	47	1 011	203	843	64	346
Zhonghuchi	51		61	474		790	172	175						287
Sanhu												686		686
Hanchihu			94		450	132	674	617	338					384
Yufeng	600													600
Candouhu					566									566
Zhuhu							565	327	168	102		231		279
Changhu	34			531		51	59	91	139		129			148
Dalianzihu			78			450	419	428	45			55	41	217
Xianghu	328										31		321	227
Meixihu							303							303
Linchonghu							39	130				72	259	125
Liuhuachi		210												210
Xieshanhu												200		200
Sanniwan				198										198
Qihu					132	66	30	197		135				112
Dawuhu								130	135					133
Zaohu	121													121
Junshanhu				114										114
Nanhu			88					100						94
Donghu	92													92
Xihu (in Jinxian county)	91													91
Beiganghu		90												90
Luojiaohu	90													90
Zhushihu	86													86
Nanshanhu		85											56	71
Zhanbeihu			36									80		58
Linghu				62										62
Meixiihu												56		56
Nanjianghu			46		55								30	44
Changhuchi													54	54
Chenjiahu								37						37
Taibohu				32										32
Xihu (in Duchang)								30						30

The lack of data in some years indicates that the numbers of oriental white storks in corresponding lakes were less than 1% of the global population number.

**Figure 3:**
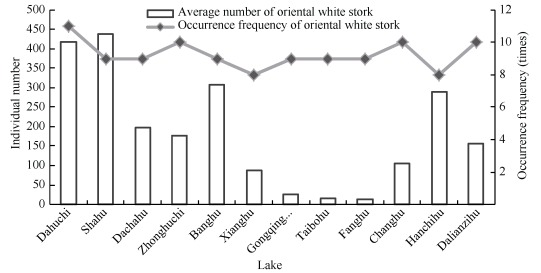
Occurrence frequencies and average numbers of wintering oriental white storks at lakes where storks were recorded more than six times between 1998 and 2011

We determined that 12-23 lakes (17 on average) were used by oriental white storks each year. Storks were found more than eight times in 12 lakes in 13 surveys (Figure 3). Among these lakes, oriental white storks were found in Dahuchi 11 times in 13 surveys, with an annual average population of 418±157. However, the individual number in Dahuchi changed drastically. For example, only five storks were observed in 2006, but 1 559 were observed in 2011. The maximum average number of oriental white storks was found in Shahu (437±208). Taibohu, Fanghu, and Nanhu (in Gongqingcheng District) were used by the storks in nine years of the study period, but the average flock sizes were only 26±13, 15±3, and 11±2, respectively. 

**Figure 4:**
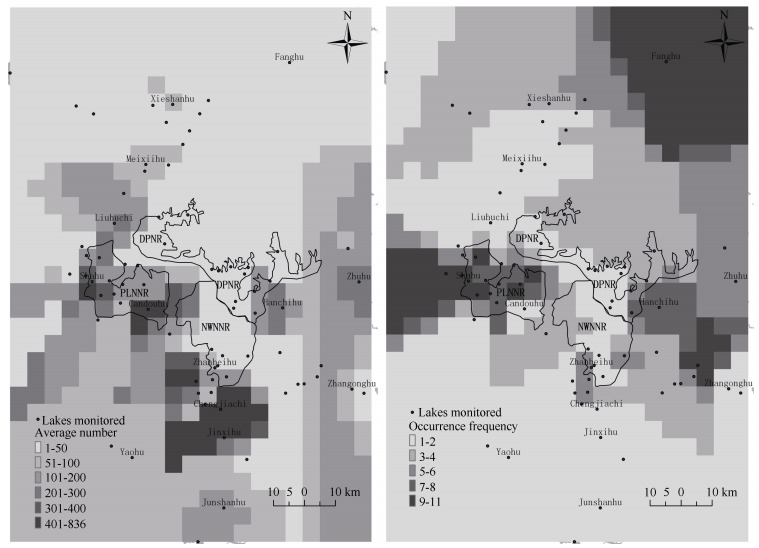
Distribution of wintering oriental white storks in Poyang Lake based on (a) average annual number and (b) occurrence frequencies

The storks showed a clustered distribution pattern at the inner lakes of Poyang Lake wetland (Figure 4a). In winter, the stork population was generally clumped on a few lakes, with a small proportion scattered across other lakes. A large proportion of the Poyang Lake population was observed on Shahu, Dahuchi, Banghu, and Hanchihu lakes, with average numbers of 418±157, 437±208, 308±123, and 290±95, respectively; large flock sizes were observed at the first three of these lakes. For example, in winter 2008, 60.2% of the oriental white stork population wintering in Poyang Lake was observed at Banghu. In every year of the study period, the largest flock sizes were found on just three lakes, where the total number of storks could be as high as 74.0%±3.6% of the overall population wintering in Poyang Lake. In winter 2008, the total number of oriental white storks on Dahuchi, Banghu, and Qihu accounted for 90.2% of the entire population at Poyang Lake. According to the occurrence frequencies of oriental white storks at these lakes, distribution across the lakes was uneven and clustered (Figure 4b). There was a significant positive correlation between occurrence frequency and average number of storks on the lakes (r=0.274, *P*=0.049, *n*=52).

**Figure 5:**
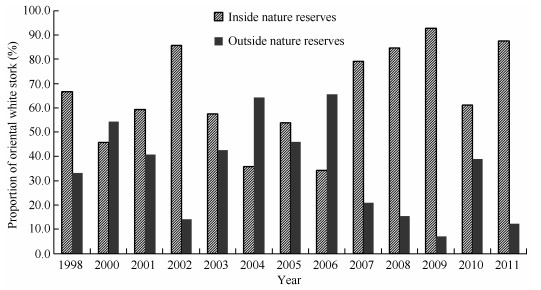
Distribution of wintering oriental white storks inside and outside nature reserves within Poyang Lake between 1998 and 2011

In Poyang Lake, most lakes of importance to the storks were found within nature reserves. The average number of storks distributed in PLNNR, NWNNR, and DPNR was 1 428±217, accounting for 64.9%±5.5% of the total population wintering in Poyang Lake (Figure 5). Among these nature reserves, PLNNR possessed the highest number of storks, which corresponded to 55.7%±5.1% of the total population. The number of storks wintering in PLNNR increased significantly after 2007, and was normally more than 58.5% of the total population in subsequent years. In 2011, the number of storks in PLNNR was 87.6%. Accordingly, the number of storks outside the nature reserves tended to decrease after 2004 (Figure 5). In 2004 and 2006, 64.3% and 65.7% of the total Poyang Lake oriental white stork population, respectively, was found outside the nature reserves. However, the numbers decreased to 7.2% during 2007-2009, and then increased to 38.9% in 2010. 

The storks used lakes within the nature reserves significantly more often (3.5±0.7, *n*=33) than those outside the nature reserves (2.5±0.4, *n*=44) (Mann-Whitney *U*=209.0, *P*=0.028). The average flock size within the nature reserves (84±26 individuals, *n*=33) was significantly larger than that outside the reserves (77±25 individuals, *n*=44) (Mann-Whitney *U*=159.0, *P*=0.002). Lakes within national nature reserves were used significantly more often (5.8±0.9 times) than those within the provincial nature reserves (0.8±0.3 times) (Mann-Whitney *U*=29.5, *P*=0.000) and those outside the nature reserves (2.4±0.4 times) (Mann-Whitney *U*=189.0, *P*=0.001). Moreover, average flock size on lakes within national nature reserves (153±40 individuals) was significantly larger than that within the provincial nature reserves (2±1 individuals) (Mann-Whitney *U*=17.5, *P*=0.000) and that outside the nature reserves (77±18 individuals) (Mann-Whitney *U*=234.0, *P*=0.011). However, the storks inhabited lakes within the provincial nature reserves significantly less often than they inhabited lakes outside the nature reserves (Mann-Whitney *U*=209.0, *P*=0.028), and a significantly smaller flock size was found on lakes within the provincial nature reserves than on lakes outside the nature reserves (Mann-Whitney *U*=159.0, *P*=0.002).

## DISCUSSION

### Population number

Oriental white storks mainly winter in the middle and lower reaches of the Yangtze River floodplain, with Poyang Lake possessing the largest wintering population in the world (Wu, 2002). Therefore, the fluctuations of stork populations in Poyang Lake are a good reflection of its global population dynamics. With only about 3 000 oriental white storks globally, this species is considered endangered (BirdLife International, 2013). We found that the number of storks in Poyang Lake in 2004, 2005, 2010, and 2011 exceeded the highest-ever estimated global population. The number in 2005 reached 3 789, and was approximately 3 400 in 2010 and 2011. This might be because of population increase or previous underestimation of population size. Zhu et al. (2012) observed 4 052 storks in December 2011, which was the largest recorded number of storks in Poyang Lake. The tendency for population growth in PLNNR between 1983 and 2011 also provides evidence toward an overall population increase. One reason for this increase might be the better protection of its habitats and decrease in illegal hunting when these habitats were enclosed in nature reserves over the last 20 years (An et al., 2007; Su et al., 2000). Another reason could be that many storks were forced to winter in Poyang Lake due to habitat deterioration of other wetlands in the middle and lower reaches of the Yangtze River floodplain (Cui et al., 2013; Liu et al., 2013). Although the population has increased obviously in recent years, it has shown extreme annual fluctuations. There were 2 832 individuals observed in 1998, but only 838 in 2008, accounting for 29.6% of the population size observed 10 years previously. The population then showed exponential growth after 2000, rising to 3 789 in 2005. However, this was again followed by a drastic reduction after 2006, and by 2009 only 430 individuals were found. In 2009, our field survey was carried out at the end of winter, when many storks may have left Poyang Lake and started their migration toward breeding grounds, thus resulting in an underestimation of the stork population for that year. 

A satellite tracking study found that oriental white storks sometimes move hundreds of kilometers over wintering ground (Van den Bossche et al., 2001). They can move between Poyang Lake in Jiangxi Province and Shengjin Lake in Anhui Province, and use an extensive area in winter (Wu et al., 2001). In our study, the field survey was implemented within one day to ensure synchronization. Nevertheless, some storks may also disperse among sub-lakes of Poyang Lake or among lakes in the middle and lower reaches of the Yangtze River floodplain, which could result in a biased estimated population. Consequently, undertaking further synchronization surveys is necessary to accurately estimate the wintering population of the oriental white stork. In addition, independent surveys conducted by other organizations should be used as references. For example, regular monthly waterbird monitoring conducted by the PLNNR could help improve the accuracy of population estimates.

Another reason for the population fluctuation might also lie in the high mortality of oriental white storks during migration. Earlier studies found that *Ciconia ciconia*, a sibling species of oriental white stork, has a high rate of mortality (52%-74%) during migration in the first winter (Van den Bossche et al., 2002), and while adult mortality rates decrease, they can remain as high as 25%-50%. Satellite tracking has also revealed that the mortality rate of oriental white storks is higher in wintering grounds and during migration (Van den Bossche et al., 2001), with five out of six storks failing to reach adulthood and perishing at stopover or wintering grounds. In wintering areas, storks can be injured by ice forming at night, even in winters without extreme low temperatures (Yu, 2013). Thus, high mortality during migration or on wintering grounds might result in the obvious fluctuations of the oriental white stork population. At the same time, factors such as deforestation, spring fire, reclamation of wetland, and overfishing also seriously threaten the survival of storks in breeding areas (BirdLife International, 2013). Therefore, it is not surprising that dramatic fluctuations in the oriental white stork population on Poyang Lake were observed within the study period. 

### Population distribution and *in-situ* conservation

Most of the important wintering lakes for oriental white storks were enclosed in nature reserves within Poyang Lake. Over the past 13 years, an average of 64.9%±5.5% of the Poyang Lake population has been observed within nature reserves. The storks were also commonly found clumped on only a few lakes. The highest occurrence frequencies and largest flock sizes were found on Shahu, Dahuchi, Banghu, and Hanchihu lakes located in PLNNR, indicating its critical importance for wintering storks. The average number of storks that wintered in PLNNR was 55.7%±5.1% of the total. In 2011, the proportion of storks in PLNNR rose to 87.6% of the total population. The clumped distribution pattern might result from less human disturbance and more suitable water depth in PLNNR. With the establishment of nature reserves such as NWNNR and DPNR in recent years, an increasing number of lakes of importance to the oriental white stork have been encompassed by nature reserves. Lakes such as Changhu, Zhanbeihu, and Sanniwan in NWNNR, and Xihu in DPNR, were observed with more than 1% of the global population of oriental white storks. 

PLNNR, NWNNR, and DPNR are located adjacent to each other. All reserves have undergone rapid development over the past 10 years. They are well-resourced and fully equipped with convenient transportation, advanced communication, and professional staff. We found that storks occurred significantly more often and aggregated in larger flocks at lakes within PLNNR and NWNNR than lakes outside these reserves. This is likely a reflection of less human disturbance and wetland degradation and more suitable lakes for wintering storks. DPNR covers the main water body of Poyang Lake and has large areas of deep water, which lowers its suitability as wading bird habitat; therefore, storks within this area were smaller in flock size and at lower frequency than those found in other lakes.

The main human activity that potentially affects the oriental white stork in Poyang Lake is traditional fishery operation. This unique mode of fishing has been popular in Poyang Lake for many hundreds of years. Local fisherman build short dams to entrench dish-shaped lakes in autumn to reserve as many fish as possible; at the start of winter, fishermen dig drainage ditches to drain off lake water and install fish pots in the opening of the ditch to catch fish, which leads to a gradual reduction in the water level until the lake dries up (Guo et al., 2014). The available habitats for the oriental white stork thus change dramatically and the storks are forced to seek new suitable habitats (Liu et al., 2011). Traditionally, administrators of all nature reserves, from national to county level, have been unable to interfere in decisions regarding water management, which have been entirely governed by fishery production to secure maximum economic benefit. Nowadays, how to harmonize traditional fishery production with the conservation of large wading birds within Poyang Lake is an issue that requires urgent resolution. Therefore, we propose that a proper ecological compensation system be established to ease the conflict between waterbird conservation and utilization of fishery resources. Given the remarkable spread of oriental white storks across the wintering grounds of this lake wetland, we also suggest that a monitoring and protection network for oriental white storks be given high priority as part of the conservation of large wading birds in the middle and lower reaches of the Yangtze River of China.
